# Oropharyngeal squamous cell carcinoma with known human papillomavirus status treated with definitive chemoradiotherapy: patterns of failure and toxicity outcomes

**DOI:** 10.1186/1748-717X-8-174

**Published:** 2013-07-09

**Authors:** Trevor J Bledsoe, Anisha R Noble, Grant K Hunter, Lisa A Rybicki, Aaron Hoschar, Deborah J Chute, Jerrold P Saxton, John F Greskovich, David J Adelstein, Shlomo A Koyfman

**Affiliations:** 1 Case Western Reserve University, School of Medicine, Cleveland, OH, USA; 2 Department of Radiation Oncology, Taussig Cancer Institute, Cleveland Clinic, Cleveland, OH, USA; 3 Department of Quantitative Health Sciences, Lerner Research Institute, Cleveland, OH, USA; 4 Department of Anatomic Pathology, Pathology and Laboratory Medicine Institute, Cleveland, OH, USA; 5 Department of Solid Tumor Oncology, Taussig Cancer Institute, Cleveland, OH, USA

**Keywords:** Oropharyngeal squamous, Human papillomavirus, Chemotherapy, Radiation, Chemoradiotherapy, Toxicity, Survival

## Abstract

**Background:**

Tumor human papillomavirus (HPV) status has emerged as one of the most powerful prognostic factors for disease control and survival in patients with oropharyngeal squamous cell carcinoma (OPSCC). We reviewed our experience in patients with OPSCC and known tumor HPV status treated with definitive chemoradiotherapy (CRT).

**Methods:**

Patients with stage III-IVb OPSCC and known tumor HPV status treated with CRT between 2006 and 2011 were identified from an IRB approved registry for this retrospective review. Outcomes were estimated using the Kaplan-Meier method and compared between HPV-positive and negative patients using the log-rank test.

**Results:**

Of the 121 pts (89% male, 93% Caucasian) included in this study, median age was 57 (range: 40–73) and median follow-up was 21 months (range: 6–63). Ninety-seven (80%) patients were HPV-positive and 24 (20%) were HPV-negative. Primary site was base of tongue (55%), tonsil (44%), and oropharyngeal wall (2%). Two year rates of locoregional recurrence (3% vs. 26%; p = 0.002), disease free survival (93% vs. 64%; p = 0.001) and overall survival (94% vs 73%; p = 0.002) were superior in HPV-positive patients, while rates of distant recurrence were similar (3% vs. 5%; p = 0.98). While acute toxicities were similar between both groups, patients with HPV-positive disease were more likely to resume a normal diet (90% vs. 65%; p = 0.017) at last follow up. Also, no HPV-positive patient required a feeding tube beyond 6 months after treatment, compared with 24% of HPV-negative patients.

**Conclusions:**

Definitive CRT produces excellent rates of disease control with minimal late toxicity for patients with HPV-positive OPSCC. Studies of OPSCC should account for tumor HPV status when identifying factors prognostic for outcome.

## Background

Recent epidemiologic studies have demonstrated that the incidence of oropharyngeal squamous cell carcinoma (OPSCC) has been steadily increasing in the United States [1,2]. This increase has largely been due to the precipitous rise of human papilloma virus (HPV) associated OPSCC, comprising approximately 70% of all oropharyngeal tumors in the U.S. [2]. The overall prevalence of oral HPV infection has recently been documented as 6.9% [1]. By contrast, the incidence of HPV-negative OPSCC, most commonly associated with tobacco and alcohol, is declining [2].

HPV-associated head and neck cancer has been shown to have a distinct set of risk factors, demographic tendencies, histologic appearances and genetic signatures and is considered to be a different disease from smoking and alcohol related HNC [3-6]. Several retrospective and prospective studies have demonstrated that the unique phenotypic and biological profile of HPV associated OPSCC translate into superior disease free and overall survival outcomes, whether treated non-operatively with definitive radiation, chemoradiotherapy, or treated primarily with surgery [5,7-11].

At our institution, we have tested oropharyngeal cancers for HPV status since 2006 using in situ hybridization. As tumor p16 expression has been found to be a useful surrogate for tumor HPV status and more sensitive at detecting all oncogenic HPV types, we began testing for p16 as well in 2009 [10]. This is the first report of our experience with tumor control outcomes, patterns of failure, and toxicity profiles in patients with locally advanced OPSCC with known HPV status treated with definitive chemoradiotherapy (CRT). We also sought to identify patient, tumor, and treatment related factors that were associated with these endpoints.

## Methods

This IRB approved retrospective review included patients with histologically confirmed stage III-IVb OPSCC with known HPV status treated with definitive chemoradiotherapy (CRT) at our institution between 1/2006 - 3/2011. Patients meeting the study criteria were identified after conducting a search of our radiation database, Mosaiq, which was queried for all patients with oropharyngeal cancer treated with RT during the years of interest. Patients treated with prior surgery or radiotherapy alone were excluded. Moderate or greater alcohol use (>3 drinks daily) and tobacco exposure data, including pack years, were collected on all patients. Patients were considered former drinkers or smokers if they quit >2 months prior to diagnosis. Patient, tumor, and treatment related factors were compared between HPV-positive and negative patients using the Wilcoxon rank sum test, Fisher’s exact test, or the Chi-square test.

Each biopsy specimen was evaluated for HPV infection with the high-risk strains of 16, 18, 31, 33 and 45. Initially, HPV status was determined using an in situ hybridization technique. Since 2009, we have also performed immunohistochemical analysis for p16 status on all patients with oropharyngeal SCC. Samples that demonstrated strong and diffuse staining in >75% of the tumor cells were considered positive. Patients with p16 expression, but negative or no HPV in situ hybridization testing were also included in the HPV-positive group.

All patients were treated definitively with CRT. Radiotherapy was delivered with once-daily fractions of 2 Gy/fx to a total dose of 70–74 Gy or in twice-daily fractions of 1.2 Gy/fx to a total dose of 74.4 Gy. Twice daily radiation was typically offered to high performing patients with T3 or T4 disease. The majority of patients were treated with a conventional 3-field approach, while more recent patients were treated with intensity-modulated radiation therapy (IMRT). All patients were administered chemotherapy. Patient treated in the earlier years were treated with 2 courses of 4-day infusions of fluorouracil (1000 mg/m2/day) and cisplatin (20 mg/m2/day) during the first and fourth weeks of radiation, as per our previous institutional standard [12]. Single agent high dose cisplatin of 100 mg/m2 on days 1, 22 and 43 of the radiation was an alternative regimen used more often in recent years. Two patients were also treated with gefitinib, an oral tyrosine kinase inhibitor in addition to cytotoxic chemotherapy as part of a phase II trial. Salvage neck dissection was reserved for patients who had metabolically active persistent neck disease evident on PET/CT three months after the completion of therapy, or in those with persistently palpable nodal masses that were of particular clinical concern.

Toxicity was scored according to the Common Terminology Criteria for Adverse Events v3.0 scale [13]. A normal diet at follow up was defined as a patient not requiring nutritional supplements or a feeding tube and having no significant dietary limitations. Routine follow-up included comprehensive multispecialty evaluation at 3 months post-treatment with imaging, typically with PET/CT. This was followed by office visits at least every 3 months for years 1–2, every 4 months for years 3–4, every 6 months for year 5, and annually thereafter. All first recurrences were histologically confirmed if possible. Failures in the oropharynx or neck were recorded as locoregional failures, while failures outside these areas were recorded as distant failures. Locoregional recurrence, distant recurrence, disease free survival (DFS) and overall survival (OS) were estimated using the Kaplan-Meier method and compared between HPV-positive and negative patients using the log-rank test. Both patients who recurred as well as patients who died with no evidence of disease were considered events when calculating DFS. Prognostic factors for outcomes were identified using Cox proportional hazards analysis. Multivariable analysis could not be done due to a small number of events. Analyses were done using SAS® software (SAS Institute, Inc., Cary, NC, USA). All statistical tests were two-sided, and p < 0.05 was used to indicate statistical significance.

## Results

One hundred twenty-one patients (108 men and 13 women) with OPSCC were included in this retrospective study. Baseline demographic and tumor related details are summarized in Table 1. Patients with HPV-negative tumors were more likely to present with advanced T stage compared with patients with HPV-positive tumors (p = < 0.001), while N stage was similar between groups (p = 0.71). The T and N distribution of the two patient groups is outlined in Table 2. Of the 121 tumors tested, 93 were found to be HPV-positive based on the results of in situ hybridization (ISH) and 4 proved ISH negative or unknown but positive for p16 expression. These four patients were included in the HPV-positive cohort. No tumors determined to be HPV-positive based on ISH were negative for p16 expression.

**Table 1:** Baseline patient and tumor characteristics by HPV status

**Variable**	**HPV-positive (97)**	**HPV-negative (24)**	**p-value**
Median age (range)	56 (40-70)	58 (45-73)	0.23
Gender			0.022
Male	90 (93%)	18 (75%)	
Female	7 (7%)	6 (25%)	
Race			0.015
Caucasian	93 (96%)	19 (79%)	
Other	4 (4%)	5 (21%)	
KPS			< 0.001
≥90	89 (92%)	14 (58%)	
<90	8 (8%)	10 (42%)	
Tobacco use			0.033
Current	14 (14%)	8 (33%)	
Former	50 (52%)	13 (54%)	
Never	33 (34%)	3 (13%)	
Median pack years (range)	10 (0-80)	30 (0-100)	< 0.001
Moderate or greater alcohol use			0.009
Ever	10 (10%)	8 (33%)	
Never	87 (90%)	16 (67%)	
Year of diagnosis (range)	2009 (2006-2011)	2009 (2007-2010)	0.26
Primary site			0.52
Base of tongue	53 (55%)	13 (54%)	
Tonsil	43 (44%)	10 (42%)	
Oropharyngeal wall	1 (1%)	1 (4%)	
Median diameter, cm (range)	2.8 (1.0-9.3)	3.9 (1.5-7.0)	0.002
Median follow-up, months (range)	20.4 (5.8-63.0)	24 (7.4-47.7)	0.47

**Table 2:** TNM Classification of patients by tumor HPV status

**N Classification**	**T Classification**			
**HPV-Positive**	**T1**	**T2**	**T3**	**T4**
**N0**			2	
**N1**		3	2	
**N2a**	6	2	1	
**N2b**	18	15	7	3
**N2c**	4	8	5	7
**N3**	3	5	3	3
**HPV-Negative**				
**N0**			1	2
**N1**			1	2
**N2a**			1	
**N2b**	2	1	1	2
**N2c**	1	0	3	3
**N3**		1	1	2

P values for differences between T stage and N stage for HPV-positive and HPV-negative groups were p = < 0.001 and p = 0.71, respectively.

The median age at diagnosis was 57, median KPS was 90, and median follow-up was 20.8 months (range 5.8-63 months). The most common primary tumor site was base of tongue (n = 66), followed by tonsil (n = 53), and oropharyngeal wall (n = 2). Patients with HPV-positive tumors were more frequently Caucasian (96% vs 79%; p = 0.015), male (93% vs 75%; p = 0.022), never smokers (34% vs 13%; p = 0.033), never moderate or greater drinkers (90% vs 67%; p = 0.009) and had KPS ≥90 (92% vs. 58%; p < 0.001). HPV-negative tumors were also more likely to be T3/T4 tumors (79% vs 34%; p < 0.001) with a larger median diameter (3.9 cm vs 2.8 cm; p = 0.002).

Treatment delivered was similar between patients with HPV-positive and HPV-negative disease (Table 3). While radiation type, median doses and fractionation were similar between the two groups, HPV-negative patients were more frequently treated with BID RT (33% vs. 12%; p = 0.027). Type of chemotherapy did not differ significantly between groups, and a similarly high proportion of patients in both groups received all planned cycles of chemotherapy (88% vs. 85%; p = 1.0). Nearly all patients completed the prescribed course of RT, with 98% of HPV-positive and 96% of HPV-negative patients completing treatment. Three patients did not complete treatment; two patients died of neutropenic fever, and one patient died of unknown cause. Two patients with HPV-positive disease received Gefitinib daily for two years in addition to cisplatin and fluorouracil.

**Table 3:** Treatment characteristics

**Variable**	**HPV-positive (97)**	**HPV-negative (24)**	**p-value**
Radiation type	65 (67%)	20 (83%)	0.12
3-field	32 (33%)	4 (17%)	
IMRT			
Frequency	85 (88%)	16 (67%)	0.027
QD	12 (12%)	8 (33%)	
BID			
Type of chemotherapy	60 (62%)	19 (79%)	0.32
C/F	37 (38%)	5 (21%)	
C			
Completed all planned chemotherapy	80 (85%)	21 (88%)	1.0

*QD* = once daily; *BID* = twice daily; *C/F* = cisplatin and 5-fluorouracil; *C* = cisplatin.

### Survival rates and patterns of failure

**Figure 1:**
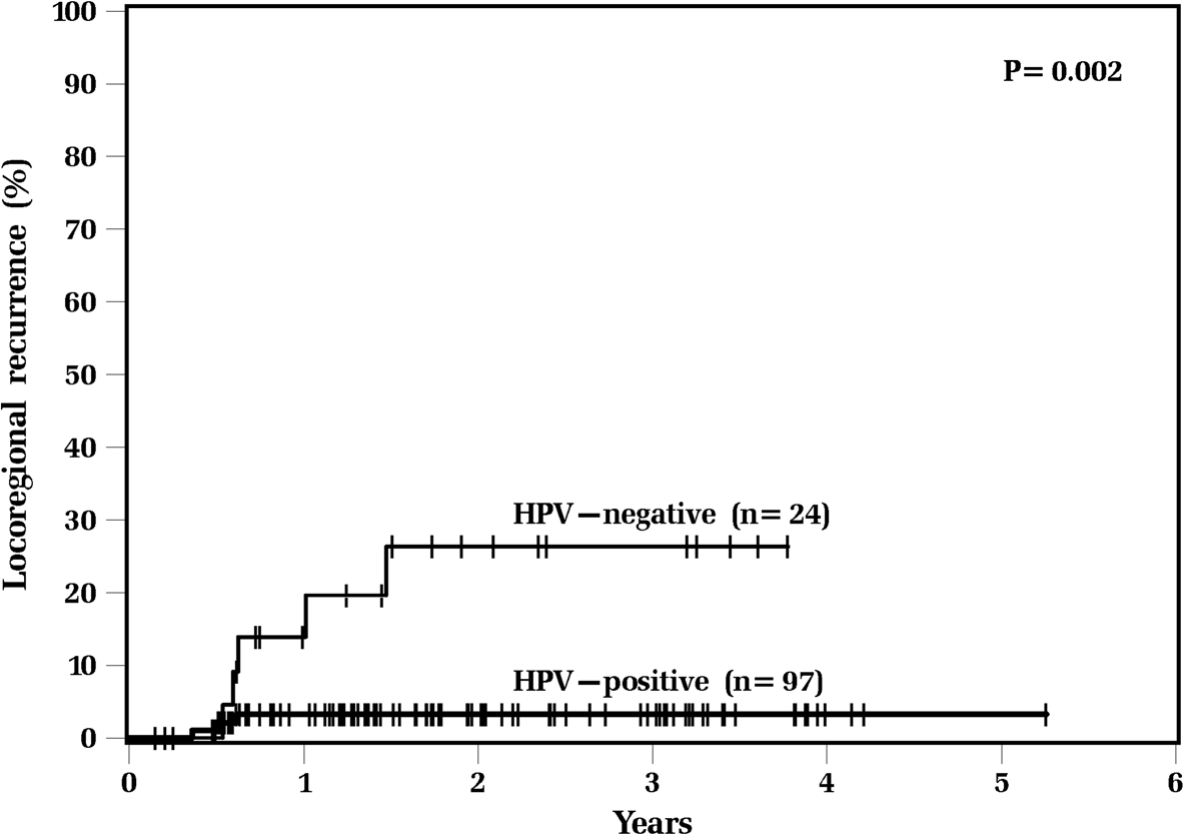
**Kaplan**-**Meier estimates of locoregional recurrence among all patients**, **according to tumor HPV status.**

**Figure 2:**
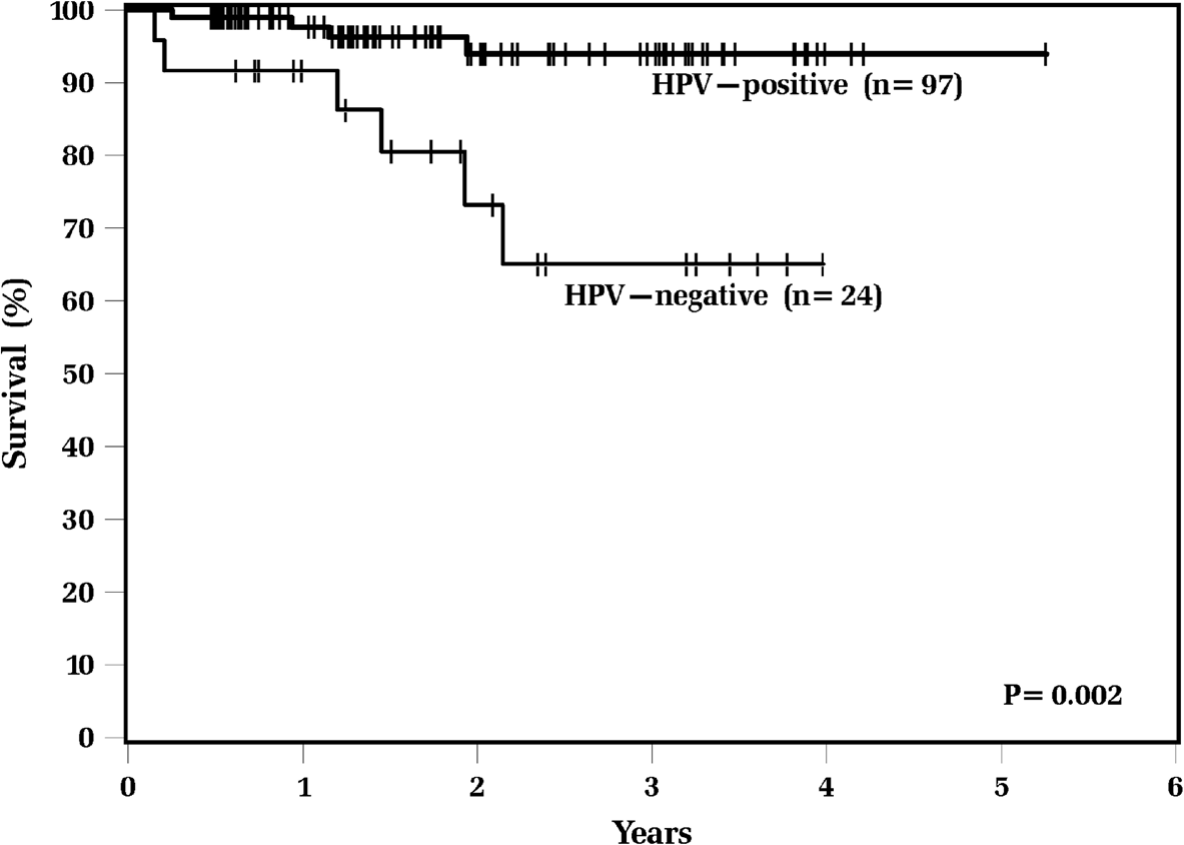
**Kaplan**-**Meier estimates of overall survival among all patients**, **according to tumor HPV status.**

Actuarial survival rates and patterns of failure data for all patients differed significantly between HPV-positive and HPV-negative tumors (Table 4). HPV-positive patients had significantly lower 2-year estimates of locoregional recurrence (3% vs. 26%; p = 0.002, Figure 1), while distant recurrence estimates were similar (3% vs. 5%; p = 0.98). HPV-positive patients had superior 2-year actuarial rates of overall survival (94% vs. 73%; p = 0.002, Figure 2) and disease free survival (93% vs. 64%; p = 0.001) compared with HPV-negative patients.

**Table 4:** Survival estimates and patterns of failure at 2 years post CRT

**Outcome at 2 years post CRT**	**HPV-positive (95% CI)**	**HPV-negative (95% CI)**	**p-value**
Locoregional recurrence	3.3% (0–6.9)	26.3% (6.0-46.6)	0.002
Distant recurrence	2.8% (0–6.9)	4.8% (0–13.9)	0.98
Overall survival	93.9% (87.9-100)	73.2% (52.3-94.2)	0.002
Disease free survival	92.7% (86.9-98.5)	63.5% (42.8-84.1)	0.001

Among patients with HPV-positive tumor status, no patients failed in the primary site. Among HPV-negative patients, all three patients with primary site recurrences died with persistent disease. Three HPV-positive patients and two HPV-negative patients failed in lymph nodes only; all five underwent salvage surgery and were disease free at last follow up. Five patients failed distantly; three died with disease and two were living with disease at last follow up. Three patients died while on or immediately after treatment—two of complications of neutropenic fever and one from an unknown cause. One patient died of mantle cell lymphoma. Univariate analysis identified factors commonly associated with HPV positive disease as being prognostic for improved overall survival, including HPV status, KPS, smoking status, T stage, primary tumor diameter, increasing duration of RT, and increasing total RT dose. Due to a lack of failure events and excellent overall survival, multivariate analysis could not be performed.

### Acute and late toxicity

Acute toxicity rates were similar between patients with HPV-positive and HPV-negative disease, including the need for feeding tubes (59% vs. 58%; p = 0.97) and rates of febrile neutropenia (29% vs. 17%; p = 0.23). Rates of radiation dermatitis (grades 1–4), pain during treatment (grades 1–3), and pain at 90 days after completing treatment (grades 0–2) were also similar with p values of 0.62, 0.57, and 0.55, respectively. Late toxicity rates differed significantly between the two groups (Table 5). Six months after the completion of RT, not a single HPV-positive patient required a feeding tube, compared to 24% of the HPV-negative cohort (p < 0.001). More HPV-positive patients had resumed a normal diet, without the need for protein supplementation, compared to the HPV-negative population (90% vs. 65%; p = 0.017). HPV-positive patients also had a significantly lower rate of trismus (grade ≥ 2) than those in the HPV-negative cohort (1% vs. 24%; p = 0.002), while rates of late grade ≥ 2 xerostomia and dysphagia were comparable (Table 5). Among the 14 HPV-positive patients who experienced grade ≥ 2 dysphagia, 8 patients had recovered, 3 had persistent dysphagia, and 3 were unknown at last follow-up. Among the five HPV-negative patients, one patient had recovered, three had persistent dysphagia, and one was unknown at last follow-up.

**Table 5:** **Late toxicity** (>**90 days post CRT**) **by HPV status**

**Late toxicity**	**HPV-positive (86)**	**HPV-negative (17)**	**p**-**value**
Need for feeding tube 6 months post-RT	0 (0%)	4 (24%)	<0.001
Normal diet at last follow up	77 (90%)	11 (65%)	0.017
Xerostomia grade ≥ 2	69 (80%)	13 (77%)	0.76
Dysphagia grade ≥ 2	14 (16%)	5 (29%)	0.20
Trismus grade ≥ 2	1 (1%)	4 (24%)	0.002

## Discussion

We have presented a large retrospective review of OPSCC outcomes stratified by known HPV/p16 status. Our findings are consistent with the mounting retrospective and prospective data that demonstrate a strong correlation between HPV-positive tumor status and improved overall survival, disease free survival, and recurrence. A review and meta-analysis summarizing such studies documented that 17/23 studies demonstrated improved outcomes for HPV-positive disease [9].

The results reported in the current study are very similar to those reported in the recent publication from Ang and colleagues summarizing the results of patients with OPSCC treated on the RTOG 0129 protocol [5]. Locoregional disease recurrence rates were significantly better among HPV-positive patients, while rates of distant failure were similar. HPV-negative disease tended to have higher T stages, which is why they were more commonly treated with twice-daily radiation per our institutional standard. Despite this approach, their locoregional control and survival were inferior. Importantly, in our series, all patients with regional only failure were successfully salvaged surgically. This emphasizes the importance of close follow up and early salvage surgery for persistent or recurrent disease in these patients. The suboptimal control rates in HPV-negative patients underscores the fact that this disease is biologically distinct, likely requiring distinct treatment approaches and intensification strategies to achieve improved tumor control outcomes.

In the subset analysis of oropharyngeal cancer patients in RTOG 0129, Ang and colleagues used HPV status, >10 smoking pack years, and tumor and nodal stage to classify patients into three categories with respect to overall survival. In the current study, the low overall failure rate among patients with HPV-positive disease precluded meaningful breakdown of these patients by smoking status. We plan to further investigate the impact of smoking on patients with HPV-positive disease in an update to this study with more patients. While this classification system of Ang and colleagues incorporates several important prognostic factors in addition to HPV status, several recently published studies have shown a number of other factors including metabolic tumor volume, presence of low lying neck disease, and the use of intensity modulated radiotherapy (IMRT) enabling a simultaneous integrated boost technique, to be associated with improved outcomes in OPSCC. However, none of these recent studies account for tumor HPV status in their analysis [14-18]. Additional investigation is needed to determine whether these and other variables are prognostic of outcomes independent of tumor HPV status, and as such, future OPSCC studies must include tumor HPV status for meaningful analysis.

As survivorship improves after chemoradiotherapy, late toxicity and its impact on long term quality of life is becoming a primary outcome of interest in these patients. Our experience, albeit with early follow up, demonstrates excellent functional outcomes overall, especially in the HPV-positive group of patients. Long term feeding tube requirements are nonexistent in this group by six months post treatment, and even in the HPV negative population, only one patient was still feeding tube dependent after one year. In addition, the overwhelming majority of patients resume a normal diet at last follow up with moderate rates of long term dysphagia and trismus. Overall, compared to a recent review of RTOG studies which revealed >40% late severe toxicity in patients treated with definitive chemoradiotherapy, our patients experienced markedly lower rates of late toxicity with less severity[19]. These results also compare favorably to outcomes in the minimally invasive surgical series [20]. This may be due to a variety of reasons, including: differences in patient and tumor characteristics, better imaging, novel planning techniques – although the majority of patients in out cohort were not treated with IMRT-, and improved supportive care. Also, patients with HPV-associated tumors may manifest less late effects than HPV-negative patients due to this group generally being younger, higher performing, with less tobacco and alcohol exposure and fewer comorbidities. These favorable late effect profiles need to be considered as new clinical trials are designed.

The present study has several limitations. It is a retrospective cohort study, with inherent selection and recall biases. The follow up was also limited and may have led to an underestimation of treatment failures and toxicity. Retrospective collection of late toxicity data is often difficult and may suffer from recall bias as well. Some heterogeneity was observed between treatment arms, such as the use of twice daily radiation, which presumably was driven by more frequent T3/4 disease in the HPV negative cohort. Due to the size of the cohort and lack of failures, meaningful multivaraiate analysis to identify prognostic factors other than HPV status was not possible.

## Conclusion

Definitive CRT produces excellent rates of disease control with minimal late toxicity for patients with HPV-positive OPSCC. Patients with HPV-negative disease have inferior locoregional control and increased late morbidity after CRT. Future studies are needed to clarify the relationship between HPV status and other prognostic factors, and to optimize treatment paradigms for HPV-positive vs. HPV-negative disease.

## Competing interests

All authors have no competing financial or non-financial interests.

## Authors’ contributions

TB participated in all aspects of the study. AN assisted in data acquisition and revision of the manuscript. GH assisted in study conception and design and revision of manuscript. LR provided analysis and interpretation of data and assisted in the revision of the manuscript. AH, DC, JP, JF, and DA assisted in study conception and design, analysis and interpretation of data, and assisted in manuscript revision. SA participated in all aspects of the study. All authors read and approved the final manuscript.

## Authors’ information

SA treats a large number of patients with head and neck cancer and is a member of the Radiation Therapy Oncology Group (RTOG) Head and Neck Cancer Committee. DA has been a principle investigator for multiple RTOG studies and is also a member of the RTOG Head and Neck Cancer Committee.
